# iPSC‐Derived iNK Progenitors Engraft and Generate NK Cells in Unconditioned and Autologous Immune Humanized Mice

**DOI:** 10.1111/cpr.70261

**Published:** 2026-07-10

**Authors:** Min Zhang, Zhiqian Wang, Yanhong Liu, Leqiang Zhang, Fangxiao Hu, Chengxiang Xia, Mengyun Zhang, Tongjie Wang, Qitong Weng, Hanmeng Qi, Yanping Zhu, Lijuan Liu, Dehao Huang, Hong‐Hu Zhu, Jinyong Wang

**Affiliations:** ^1^ Chinese Institutes for Medical Research Beijing China; ^2^ The Beijing Key Laboratory of Tumor Resistance Mechanism and Clinical Translation, Affiliated with the Chinese Institutes for Medical Research Beijing China; ^3^ State Key Laboratory of Organ Regeneration and Reconstruction, Institute of Zoology Chinese Academy of Sciences Beijing China; ^4^ University of Chinese Academy of Sciences Beijing China; ^5^ Beijing Institute for Stem Cell and Regenerative Medicine Beijing China; ^6^ Department of Hematology, Beijing Chao‐Yang Hospital Capital Medical University Beijing China

## Abstract

NK cells exhibit inherently short persistence in vivo. Allo‐NK cells trigger host immune rejection in immune‐competent patients. Lymphodepletion is a conventional approach for mitigating allogeneic rejection of therapeutic NK cells, which brings host immune suppression and infection risks. To address these problems, we test the concept of engrafting iNK progenitor (iNKP) cells derived from human induced pluripotent stem cells (iPSC) to achieve generating iNK cells in vivo in unconditioned host‐immune humanised mice without the requirement for prior lymphodepleting chemotherapy or total body irradiation. Our results showed that a single low‐dose infusion of iNKP cells successfully engrafted and continuously produced mature iNK cells in unconditioned B‐NDG hIL15 mice. Furthermore, the iPSC‐derived iNKP cells survived in the presence of autologous PBMC‐humanised mice and generated mature iNK cells. This study provides evidence that autologous iPSC‐derived iNKP cells have the application potential for treating the same individual without the preceding necessity of lymphodepletion.

## Graphical abstract

Infusion of CXCR4‐expressing iNK progenitor (iNKP) cells derived from iPSCs can continuously generate iNK cells in an unconditioned and autologous PBMC humanised hIL15 mouse model. iNKP cell therapy has the potential to treat patients without the requirement for prior lymphodepletion.

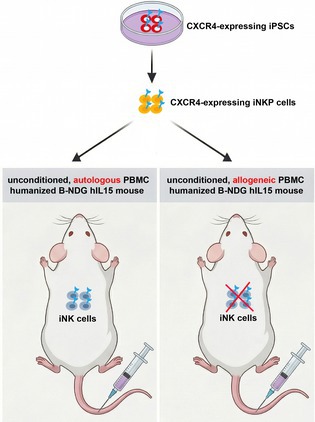

## 1 Introduction

NK cell therapy shows favourable safety features besides the universal anti‐tumour, anti‐viral infection and anti‐senescence evidence [1, 2, 3, 4]. However, current NK sources, mainly donor peripheral blood and umbilical cord blood, impose clinical limitations. Without additional modifications, allo‐NK cells show limited proliferation capability in vivo. Allo‐NK cells are susceptible to host immune rejection, which causes immediate clearance in patients. Consequently, high doses and repeated infusions of tissue‐derived NK cells are applied to enhance efficacy in treating diseases, significantly increasing costs and reducing feasibility [5, 6, 7].

A de novo iNK progenitor (iNKP) cell therapy strategy to overcome the short‐term persistence of NK cells has been developed. When combined with chemotherapy, CAR‐iNKP cells engrafted and generated CAR‐iNK cells in vivo, which persist over 1 month during the battle against human B‐ALL and T‐ALL disease models. CAR‐iNKP therapy effectively eliminates minimal residual disease and significantly reduces tumour relapse [8].

Conventional tumour immunotherapies typically require preceding chemotherapy, which immediately reduces tumour burden and facilitates the survival of adoptive immune cells via reducing host rejection [9]. However, chemotherapy also brings pan‐cytopenia, immune suppression, reactive myelopoiesis and infection [10, 11]. It remains unknown whether iNKP cells require conditioning for engraftment.

Induced pluripotent stem cell (iPSC) is a milestone for regenerative medicine [12, 13, 14]. Mouse iPSC‐derived teratomas are specifically recognised and eliminated by immune‐competent syngeneic hosts, which provides experimental evidence that autologous iPSCs still have immunogenicity [15]. Conversely, other studies have reported that most functional cells derived from autologous iPSCs do not elicit immune rejection [16, 17]. Thus, a critical question remains whether iPSC‐derived iNKP cells are immune‐compatible in an autologous immune‐competent condition in vivo.

This study attempts to simulate a clinically relevant scenario by investigating the engraftment kinetics, differentiation and maturation and immune compatibility of iNKP cell therapy in unconditioned and autologous peripheral blood mononuclear cells humanised animal models. Our findings reveal the application potential of iNKP cell therapy under unconditioned and immune‐competent conditions.

## 2 Materials and Methods

### 2.1 Peripheral Blood Cell Collection and Isolation

Peripheral blood was collected from healthy donors. Peripheral blood mononuclear cells (PBMCs) were isolated via Lymphoprep (Serumwerk Bernburg AG) density gradient centrifugation to remove erythrocytes and granulocytes, followed by ACK lysis buffer (Gibco) treatment for residual erythrocyte clearance. PBMCs were used to generate iPSCs, NK cells and T cells. HLA class I typing of donors is presented in Table S1.

### 2.2 iPSC Line Generation and Engineering

A human iPSC line was generated from healthy donor‐derived PBMCs using the HapCult Episomal Reprogramming Kit (Precision BioMedicals Co. Ltd). The *CXCR4* coding sequence was cloned into the PiggyBac vector PB530A‐2 (SBI) to generate the recombinant construct. This CXCR4‐expressing vector and the transposase vector were electroporated into iPSCs using the Celetrix Electroporator EX+ (Model 11‐0106). At Day 7 post‐electroporation, CXCR4^+^ iPSCs were isolated by two rounds of FACS to establish the R4‐iPSC line. Subsequently, the luciferase‐P2A‐puromycin cassette was inserted into PB530A‐2 and electroporated into R4‐iPSCs. Puromycin‐resistant clones were expanded to generate the R4‐iPSC‐luci line. All iPSC lines were cultured in Essential 8 medium (Gibco) on vitronectin‐coated plates (Gibco), and maintained at 37°C with 5% CO_2_ in a humidified incubator.

### 2.3 PBMC‐Derived NK Cell and T Cell Expansion

The PBMC‐NK cells isolated by the NK Cell Isolation Kit (Miltenyi Biotec) were stimulated with K562‐mIL‐21 cells (Hangzhou Zhongying Biomedical Technology Co. Ltd), 5% Supergrow Supplement (DAKEWE) and recombinant human IL‐2 (Miltenyi Biotec, 200 U/mL) in KBM581 Medium (Corning). After 12 days of expansion, NK cells were harvested. T cells derived from PBMCs were expanded using the Human T Cell Activation/Expansion Kit (Miltenyi Biotec) and harvested after 2 weeks.

### 2.4 iNKP Cell Generation In Vitro

The method to induce iPSC‐R4‐iNKP‐luci cells from R4‐iPSC‐luci cell line was as previously described [8, 18]. Briefly, R4‐iPSC‐luci cells were subjected to a 2‐day monolayer induction to induce highly purified R4‐iPSC‐luci lateral plate mesoderm (iPSC‐R4‐iLPM‐luci) cells. Subsequently, 2 × 10^4^ iPSC‐R4‐iLPM‐luci cells and 5 × 10^5^ OP9 cells were assembled into organoid aggregates and seeded into the transwell (Guangzhou Jet Bio‐Filtration Co. Ltd.), establishing an air‐liquid interface for iPSC‐R4‐iNKP‐luci cell differentiation. iPSC‐R4‐iNKP‐luci cells with the phenotype of CD45^+^CD56^−^ CD34^+^CD7^+/−^ were sorted on Day 19.

### 2.5 iNK Cell Generation In Vivo

iPSC‐R4‐iNKP‐luci cells (1.0 × 10^5^ or 2.0 × 10^5^ cells/mouse) were intravenously infused into unconditioned B‐NDG or B‐NDG hIL15 mice. Bioluminescence imaging (BLI) using the IVIS Spectrum system (PerkinElmer) was performed on Days 14, 18, 21, 25, 30, 38, 45 and 52, while peripheral blood analysis of iPSC‐R4‐iNK‐luci cells was conducted on Days 7, 14, 21, 25, 34 and 49 post iPSC‐R4‐iNKP‐luci cell infusion.

### 2.6 Flow Cytometric Analysis and Sorting

Following dissociation with 1 mL TrypLE (Gibco) at 37°C for 3 min, the iPSC‐derived engineered cell lines were collected for sorting. R4‐iPSC line was stained with an anti‐CXCR4 antibody. For iNKP cell analysis and sorting, organoids were digested and filtered through a 70 μm strainer, and centrifuged (500*g*, 5 min). Cells were then stained with antibodies specific to the CD45^+^CD56^−^CD34^+^CD7^+/−^CXCR4^+^iPSC‐R4‐iNKP‐luci cells. To analyse iNK cells, retro‐orbital blood and various organs (bone marrow, liver, lung and spleen) were collected from B‐NDG hIL15 mice. Red blood cells were lysed with ACK buffer on ice for 10–15 min, followed by resuspension in PBS, filtration through a 70 μm strainer (Biologix), and centrifugation prior to antibody staining. iNK cells were identified as CD45^+^CD3^−^CD56^+^ CD16^+/−^CXCR4^+/−^HLA‐DR/DP/DQ^−^. Analysis was performed on a BD LSRFortessa and sorting on a BD FACSAria Fusion. Data were analysed using FlowJo v10.8.1.

### 2.7 Mice

B‐NDG (NOD.CB17‐Prkdc^scid^ Il2rg^tm1Bcgen^/Bcgen) and B‐NDG hIL15 (NOD.CB17‐Prkdc^scid^ Il2rg^tm1Bcgen^Il15^tm1(IL15)Bcgen^/Bcgen) mice (8–10 weeks, female) were purchased from Biocytogen. Mice were housed in the SPF‐grade animal facility of the Institute of Zoology, Chinese Academy of Sciences.

### 2.8 Ethics Statement

Experiments and handling of mice were conducted under the Institutional Animal Care and Use Committee of the Institute of Zoology, Chinese Academy of Sciences. The iPSC lines, as well as NK cells and T cells derived from PBMCs, were obtained from donors who provided written informed consent in accordance with approved ethical guidelines.

### 2.9 Statistics

Data are presented as mean ± SD. All quantitative analyses were performed with SPSS (version 21, IBM Corp., Armonk, NY, USA). The two‐sided unpaired Student's *t*‐test was used to compare two groups of data. Graphs were created using GraphPad Prism (8.0.1, GraphPad Software).

## 3 Results

### 3.1 Human iPSC‐Derived CXCR4‐iNKP Cells Successfully Engraft in Unconditioned hIL15 Mice

**FIGURE 1:**
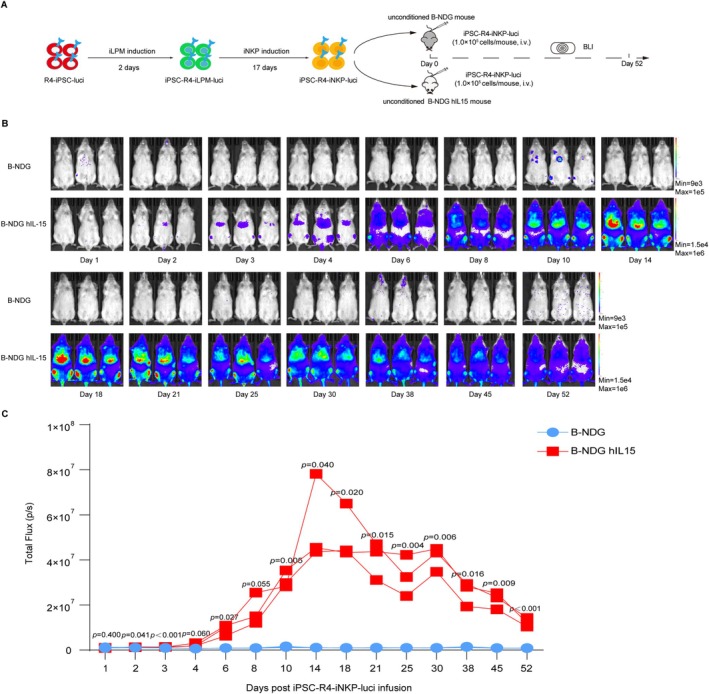
Assessment of iPSC‐R4‐iNKP‐luci cell engraftment in unconditioned B‐NDG and B‐NDG hIL15 mice. (A) Schematic diagram of generating iPSC‐R4‐iNKP‐luci cells and evaluating the distributions of these cells and their progeny in B‐NDG or B‐NDG hIL15 recipients following infusion. iPSC‐R4‐iNKP‐luci cells were intravenously infused into unconditioned B‐NDG or B‐NDG hIL15 mice. BLI was performed at the indicated time points. (B) BLI images showing the distributions of iPSC‐R4‐iNKP‐luci cells and their progeny cells in B‐NDG or B‐NDG hIL15 recipients. (C) Statistical analysis of the total flux (p/s) in (B). All data represent as mean ± SD and were analysed by two‐sided unpaired Student's *t*‐test (B‐NDG hIL15 vs. B‐NDG control). *n* = 3 biologically independent mice per group. Each line represents an individual mouse.

We previously reported that CXCR4 is critical for the homing of iNKP cells to the bone marrow [8]. To track the fate of iNKP cells in unconditioned immunodeficient mice, we generated iPSC‐R4‐iNKP‐luci cells by differentiating human pluripotent stem cells expressing the luciferase gene and the *CXCR4* gene (R4‐iPSC‐luci) via directed differentiation for 19 days. Subsequently, the sorted iPSC‐R4‐iNKP‐luci cells (1.0 × 10^5^ cells/mouse) were intravenously infused into unconditioned B‐NDG and human IL‐15‐expressing B‐NDG (B‐NDG hIL15) immunodeficient mice (Figure 1A). Quantitative BLI confirmed that iPSC‐R4‐iNKP‐luci cells achieved significant engraftment exclusively in B‐NDG hIL15 mice, with the total flux reaching a peak of approximately 8 × 10^7^ p/s at Day 14 post‐infusion (*p* = 0.040, two‐sided unpaired Student's *t*‐test). Notably, this bioluminescent signal remained statistically detectable above the baseline of B‐NDG control mice through Day 52, indicating sustained iNK persistence (Figure 1B,C). In contrast, iPSC‐R4‐iNKP‐luci cells failed to engraft in B‐NDG mice lacking human IL‐15. Collectively, these results demonstrate that iPSC‐R4‐iNKP‐luci cells achieve long‐term engraftment in unconditioned human IL‐15 humanised immunodeficient mice.

### 3.2 iNKP Cells Are Able to Generate Mature iNK Cells in Unconditioned hIL15 Mice

**FIGURE 2:**
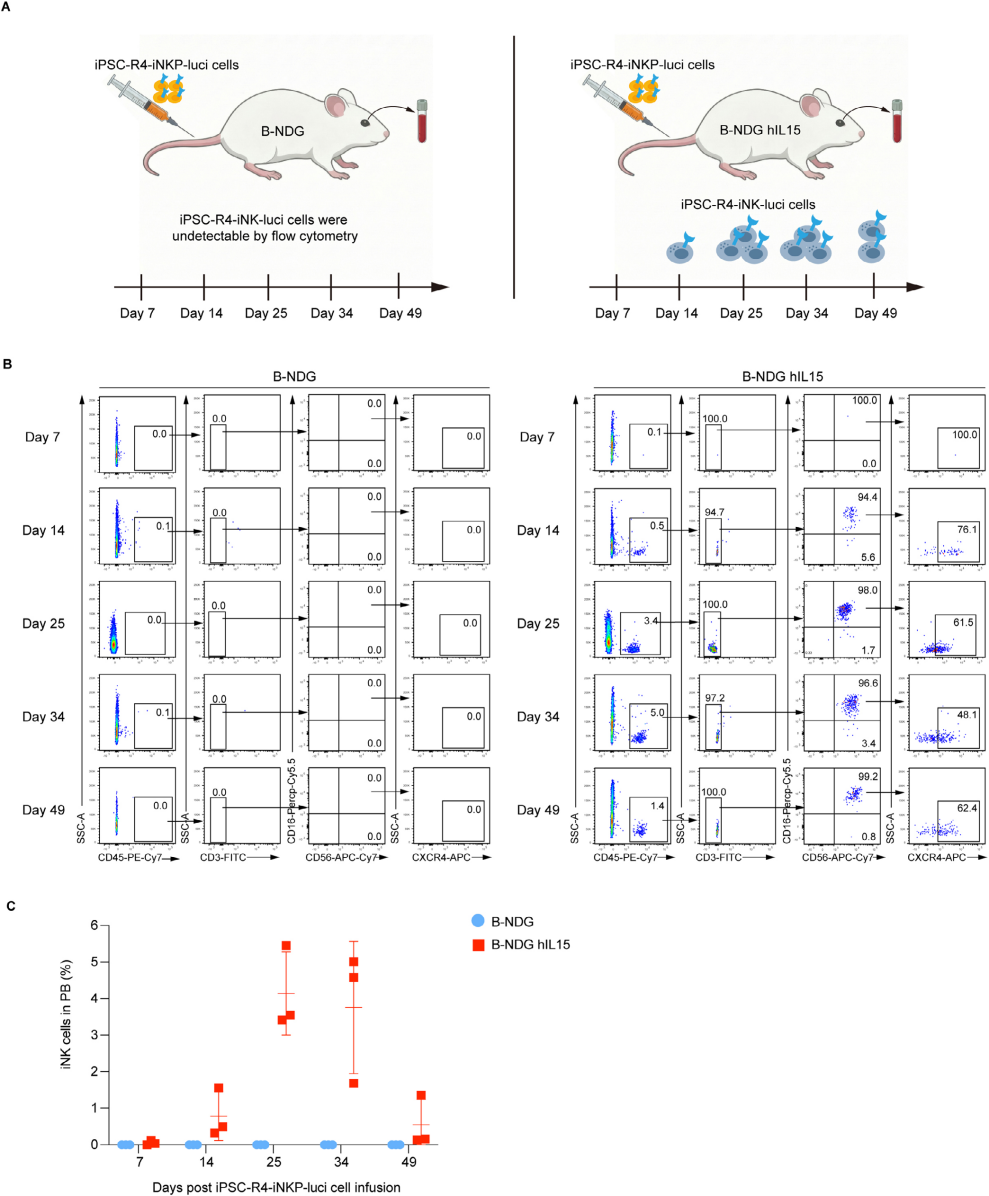
Contribution of iNK cells derived from iPSC‐R4‐iNKP‐luci cells in unconditioned B‐NDG and B‐NDG hIL15 mice. (A) Schematic diagram of detecting iPSC‐R4‐iNK‐luci cells in circulating peripheral blood (PB) at specific time points after infusion of iPSC‐R4‐iNKP‐luci cells. Flow cytometry was performed on Day 7 to Day 49. (B) Flow cytometry analysis of iPSC‐R4‐iNKP‐luci cells in PB from the B‐NDG or B‐NDG hIL15 recipients. Representative mice were shown. (C) Statistical analysis of iPSC‐R4‐iNK‐luci cells in PB of the B‐NDG or B‐NDG hIL15 recipients on Days 7, 14, 25, 34 and 49 post iPSC‐R4‐iNKP‐luci cell infusion. *n* = 3 biologically independent mice per group.

To dynamically monitor the contribution rate of iNK cells in vivo, peripheral blood was collected from unconditioned B‐NDG and B‐NDG hIL15 mice on Day 7, Day 14, Day 25, Day 34 and Day 49 after iPSC‐R4‐iNKP‐luci cell infusion (Figure 2A). Flow cytometric analysis of the peripheral blood at Day 14 revealed a distinct population of iPSC‐R4‐iNK‐luci cells exhibiting robust maturation, as evidenced by a CD16^+^ frequency exceeding 90%. The contribution rate of these cells in the circulation increased significantly, peaking between Days 25 and 34, whereas iPSC‐R4‐iNK‐luci cells remained undetectable in the hIL15‐deficient B‐NDG cohort (Figure 2B,C). These findings indicate that iPSC‐R4‐iNKP‐luci cells not only achieve engraftment and survival in unconditioned human IL‐15 humanised immunodeficient mice but also further differentiate into mature iNK cells.

### 3.3 iNKP Cells Successfully Engraft in Autologous but Not Allogeneic PBMC Humanised hIL15 Mice

**FIGURE 3:**
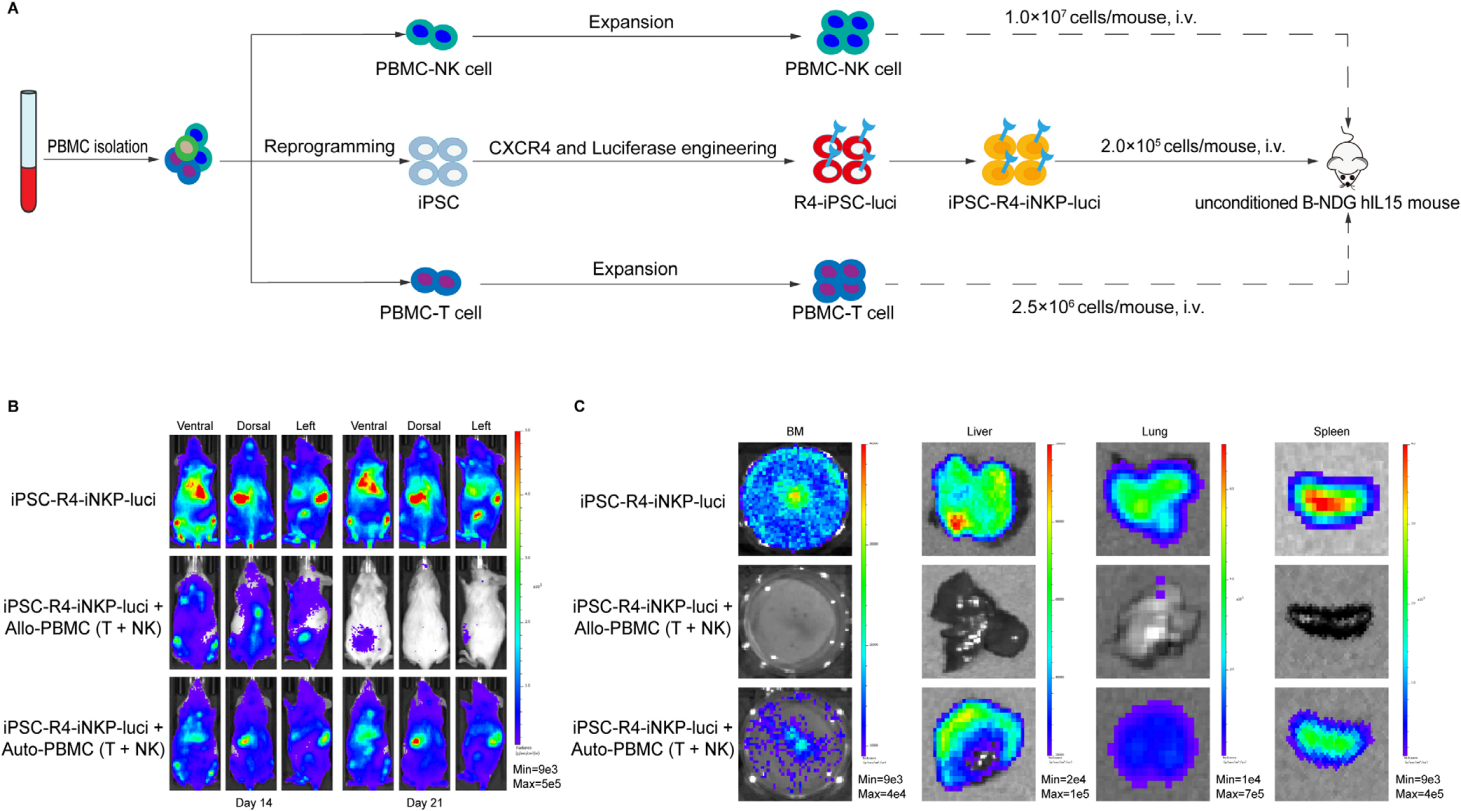
Evaluation of iPSC‐R4‐iNKP‐luci cell engraftment in a PBMC‐humanised mouse model. (A) Schematic diagram for evaluating the engraftment capability of iPSC‐induced iNKP cells under autologous immune surveillance in vivo. (B) BLI images (ventral, dorsal and left) at Day 14 and Day 21 showing the distribution of iNKP cells and their progeny cells in mice infused with: IPSC‐R4‐iNKP‐luci alone, iPSC‐R4‐iNKP‐luci + Allo‐PBMC (T + NK), or iPSC‐R4‐iNKP‐luci + Auto‐PBMC (T + NK). (C) Ex vivo BLI images of the bone marrow (BM), liver, lung and spleen isolated from B‐NDG hIL15 mice on Day 21 after iPSC‐R4‐iNKP‐luci, iPSC‐R4‐iNKP‐luci + Allo‐PBMC (T + NK), or iPSC‐R4‐iNKP‐luci + Auto‐PBMC (T + NK) cell infusion.

Considering the future scenario in clinical settings, we attempted to assess the capability of iPSC‐induced iNKP cells for iNK cell differentiation in B‐NDG hIL15 mice that received a humanised immune system constructed using the same iPSC‐donor‐derived T and NK cells (Figure 3A). In detail, we first generated iPSCs from peripheral blood mononuclear cells (PBMCs) of a healthy donor. By engineering these iPSCs to express CXCR4 and luciferase, we established a CXCR4‐iPSC‐luciferase (R4‐iPSC‐luci) cell line. Further, T cells and NK cells were isolated and expanded from both the same donor's PBMCs (autologous) and from an HLA‐mismatched healthy donor's PBMCs (allogeneic). These cells were used to reconstitute humanised immune systems in B‐NDG hIL15 mice, creating PBMC humanised models with T and NK cell compartments. Next, we differentiated the R4‐iPSC‐luci cells into iPSC‐R4‐iNKP‐luci cells. The sorted iPSC‐R4‐iNKP‐luci cells (2.0 × 10^5^ cells/mouse) were infused into unconditioned B‐NDG hIL15 mice. A subset of mice was infused with autologous in vitro‐expanded T cells (2.5 × 10^6^ cells/mouse) and NK cells (1.0 × 10^7^ cells/mouse), defining the iPSC‐R4‐iNKP‐luci + Auto‐PBMC (T + NK) group. Control groups included mice infused with only iPSC‐R4‐iNKP‐luci cells, and mice that received iPSC‐R4‐iNKP‐luci cells along with allogeneic T and NK cells (iPSC‐R4‐iNKP‐luci + Allo‐PBMC [T + NK]) (Figure 3A).

BLI on Day 14 post‐infusion revealed signals in the lung, liver, bone marrow and spleen of mice in both the iPSC‐R4‐iNKP‐luci only and the iPSC‐R4‐iNKP‐luci + Auto‐PBMC (T + NK) groups. This further demonstrated that iPSC‐R4‐iNKP‐luci cells could successfully engraft in the presence of autologous immune surveillance. By Day 21, BLI signals were nearly undetectable in all organs of the iPSC‐R4‐iNKP‐luci + Allo‐PBMC (T + NK) group, indicating complete clearance of the iPSC‐R4‐iNKP‐luci progeny cells. In contrast, fluorescence signals persisted in the organs of the iPSC‐R4‐iNKP‐luci + Auto‐PBMC (T + NK) group (Figure 3B). Subsequently, all mice were euthanized for ex vivo BLI analysis of the bone marrow, liver, lung and spleen. Ex vivo BLI confirmed the absence of fluorescence signals in all organs from the iPSC‐R4‐iNKP‐luci + Allo‐PBMC (T + NK) group, while signals were present in the other groups (Figure 3C). In summary, iPSC‐R4‐iNKP‐luci cells successfully engrafted and persisted in the autologous immune environment, whereas complete rejection occurred in the allogeneic setting.

### 3.4 iPSC‐Derived iNKP Cells Successfully Differentiate Into Mature iNK Cells in Autologous PBMC Humanised hIL15 Mice

**FIGURE 4:**
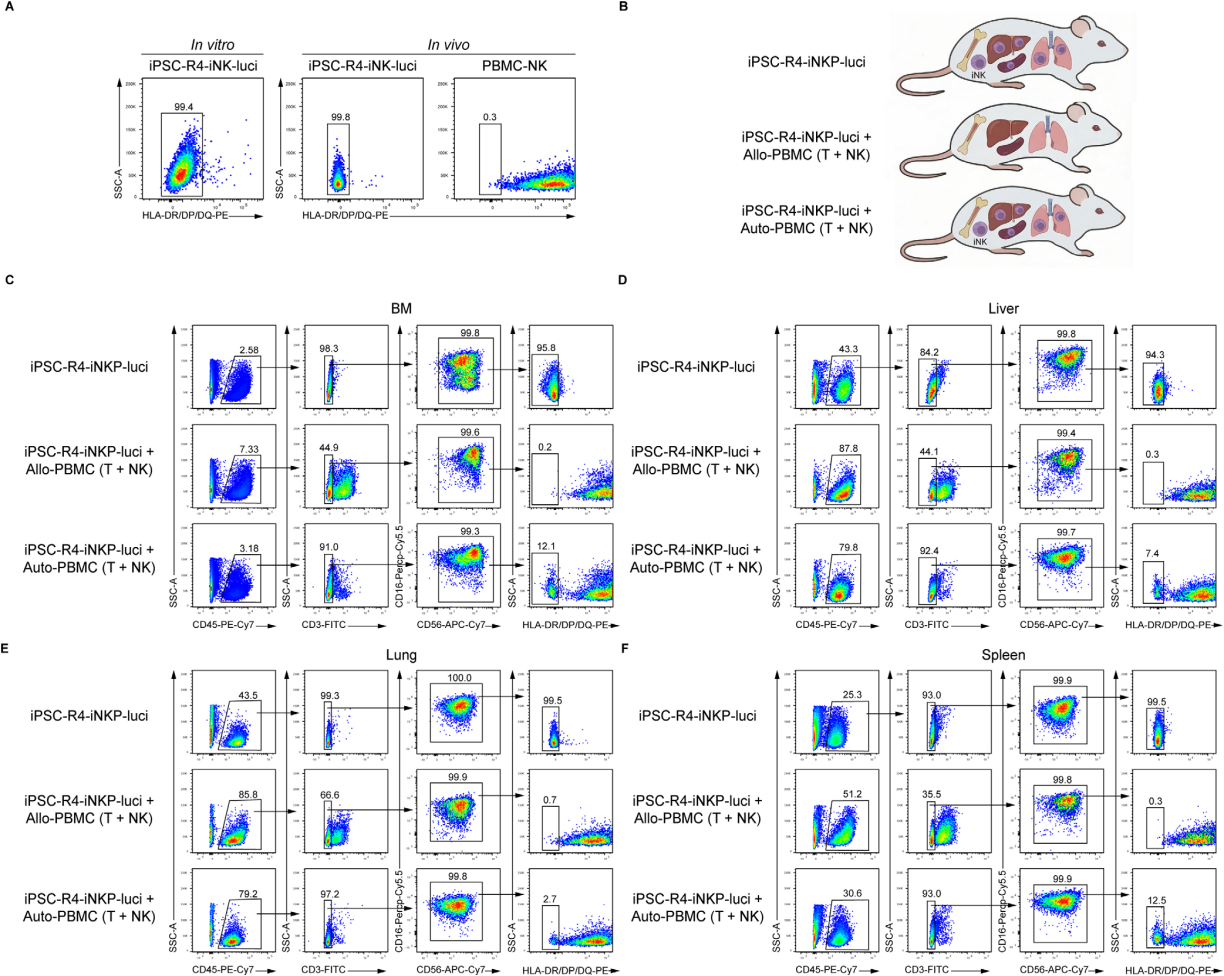
Evaluation the contribution rate of iNK cells from iPSC‐R4‐iNKP‐luci cell in a PBMC‐humanised mouse model. (A) Flow cytometry analysis of HLA‐II expression on iNK cells derived from iPSC‐R4‐iNKP‐luci cells or PBMC‐NK cells. (B‐F) Schematic diagram (B) and flow cytometry analysis of iNK cells in the BM (C), liver (D), lung (E) and spleen (F) of B‐NDG hIL15 mice infused with iPSC‐R4‐iNKP‐luci, iPSC‐R4‐iNKP‐luci + Allo‐PBMC (T + NK), or iPSC‐R4‐iNKP‐luci + Auto‐PBMC (T + NK) cells at 21 days post‐infusion. The percentages of HLA‐DR/DP/DQ‐negative iNK cells shown in the gates represent the proportion relative to total CD56^+^ NK cells.

We previously reported that human pluripotent stem cell‐derived iNK cells express negligible levels of HLA‐II molecules (HLA‐DR/DP/DQ), whereas activated/expanded primary PBMC‐NK cells express HLA‐II molecules [19]. Thus, we can distinguish the in vivo‐differentiated iNK cells from the adoptively transferred PBMC‐NK cells by flow cytometry analysis of HLA‐II molecular expressions. The results confirmed that 99.8% of iNK cells differentiated from iPSC‐R4‐iNKP‐luci cells in vivo were HLA‐II negative. In contrast, the infused PBMC‐NK cells were nearly all HLA‐II positive (Figure 4A). Thus, HLA‐II indeed can serve as a reliable marker for discriminating between iNK cells and NK cells.

Flow cytometry further demonstrated that iNK cells existing in the bone marrow, liver, lung and spleen of the iPSC‐R4‐iNKP‐luci only group were uniformly HLA‐II negative. Particularly, in the iPSC‐R4‐iNKP‐luci + Auto‐PBMC (T + NK) group, a population of HLA‐DR/DP/DQ‐negative iNK cells (2.7%–12.5%) was detected across all organs analysed. Conversely, HLA‐DR/DP/DQ‐negative iNK cells were virtually absent in all organs from the iPSC‐R4‐iNKP‐luci + Allo‐PBMC (T + NK) group (Figure 4B–F). These results demonstrate that iPSC‐R4‐iNKP cells can differentiate into mature iNK cells in vivo in the presence of a functional autologous immune system.

## 4 Discussion

The findings of this study represent a potential paradigm shift in the field of cell‐based immunotherapy. Conventional adoptive immune cell therapies are historically tethered to the necessity of conditioning regimens, such as chemotherapy or irradiation, to facilitate donor cell survival and mitigate host rejection [9]. However, these regimens introduce significant systemic toxicities, including pan‐cytopenia and increased infection risk, often precluding treatment for the most vulnerable patient populations [10, 11, 20]. Zhang et al. systematically reviewed the major toxicities associated with gastric cancer immunotherapy, pointing out that immune checkpoint inhibitors may induce autoimmune toxicities such as thyroid dysfunction and autoimmune diabetes, while CAR‐T cell therapy is often accompanied by cytokine release syndrome and neurological toxicity, which can be life‐threatening in severe cases. The clinical application of immunotherapy for treating gastric cancer is significantly hampered by side effects and toxicity [21].

Our study demonstrates that CXCR4‐expressing iNKP cells can successfully home to the bone marrow niche and differentiate into mature, circulating iNK cells in unconditioned, immune‐competent environments. This suggests a transition from a clinical model of ‘immune ablation and reconstitution’ to one of ‘immune coexistence’. By bypassing the requirement for lymphodepletion, iNKP therapy could be administered to patients with low tumour burden or those with organ dysfunctions who cannot tolerate intensive conditioning. Furthermore, the lack of conditioning may allow for theoretically unlimited repeated infusions to enhance therapeutic efficacy without cumulative toxicity.

The significance of these findings extends to the ongoing debate regarding the immunogenicity of iPSC‐derived products. While allogeneic iNKP cells were rapidly cleared by the host immune system, autologous iNKP cells successfully engrafted and matured despite the presence of active immune surveillance. This aligns with previous evidence that differentiated cells derived from syngeneic iPSCs (such as hepatocytes, endothelial cells and neuronal cells) elicit only limited immunogenicity [16]. Consequently, our results reinforce the feasibility of personalised, patient‐specific iNKP therapies as a safe and effective treatment strategy. Overcoming allogeneic immune rejection is the key to transforming iNKP cells into universal off‐the‐shelf products. Developing hypoimmunogenic iNKP cells therefore represents the next critical challenge, as achieving universality would substantially reduce the cost of iNKP cell therapy.

Despite these promising results, several limitations must be acknowledged. First, the study was conducted in humanised mouse models. While these models are sophisticated, they do not fully replicate the complexity of a human patient's immune system, particularly in the context of a ‘real‐world’ malignancy that may further alter the immune landscape. Second, our observations were limited to a 52‐day window. Long‐term studies are necessary to determine the ultimate fate of iNKP‐derived cells and to ensure that continuous production does not lead to exhaustion or unintended side effects. Third, we did not analyse whether autologous T cells or NK cells in the humanised mice became exhausted, nor did we examine whether regulatory T cells (Tregs) were induced. Future studies should employ co‐culture assays to evaluate whether iNKP or iNK cells directly affect the functional status of autologous T cells and NK cells. Furthermore, if Tregs are induced, Treg depletion studies should be performed to determine whether Tregs play a protective role in promoting iNKP engraftment in immune‐competent environments. Finally, the absolute necessity of IL‐15 for engraftment highlights that while exogenous lymphodepletion is avoidable, specific cytokine support remains essential for therapeutic success. Future research should focus on clinical validation using patient‐derived cells to confirm these ‘coexistence’ kinetics in humans.

Beyond these acknowledged limitations, one additional observation from our study warrants further discussion. The BLI signals in the “iNKP only” group appeared stronger than those in the ‘iPSC‐R4‐iNKP‐luci + Auto‐PBMC (T + NK)’ group at Day 14 and Day 21 (Figure 3B). We speculate this may be due to: (1) cytokine competition, particularly for human IL‐15, as co‐infused autologous T/NK cells also depend on IL‐15 for survival and proliferation, potentially limiting iNKP/iNK cell expansion; and (2) niche competition, where co‐infused PBMC cells occupy CXCL12‐abundant bone marrow niches, reducing iNKP homing and iNK differentiation. Importantly, this does not alter the qualitative conclusion that autologous iNKP/iNK cells can engraft in autologous PBMC‐humanised mice. Future studies will investigate this further using donors with diverse HLA typing, reduced PBMC (T + NK) doses, and in vitro co‐culture killing assays.

In summary, this study provides evidence that iNKP cells can generate iNK cells in unconditioned and autologous immune‐competent conditions. iNKP cell therapy has the potential to treat patients without the necessity of lymphodepletion or myeloablation.

## Author Contributions

Min Zhang, Zhiqian Wang and Yanhong Liu performed the core experiments and contributed equally to this work. Leqiang Zhang, Fangxiao Hu, Chengxiang Xia, Mengyun Zhang, Tongjie Wang, Qitong Weng, Hanmeng Qi, Yanping Zhu, Lijuan Liu and Dehao Huang participated in multiple experiments. Dehao Huang, Hong‐Hu Zhu and Jinyong Wang designed the project, discussed the data and wrote the manuscript. Jinyong Wang provided the final approval of the manuscript.

## Funding

This work was supported by the National Natural Science Foundation of China (82450001, 82300132, 32300676 and 82470120), the National Key R&D Program of China (2024YFA1108302) and the Noncommunicable Chronic Diseases‐National Science and Technology Major Project (2023ZD0501200).

## Conflicts of Interest

The authors declare no conflicts of interest.

## Supporting information


**Table S1:** HLA class I genotypes of the autologous and allogeneic donors.

## Data Availability

The data that support the findings of this study are available from the corresponding author upon reasonable request.
