# Supplementary material for: iPSC‐Derived iNK Progenitors Engraft and Generate NK Cells in Unconditioned and Autologous Immune Humanized Mice
Source: Cell Prolif. 2026 Jul 10:e70261. Online ahead of print. doi: 10.1111/cpr.70261 (PMC13354730; doi:10.1111/cpr.70261)
Supplement: Supplementary file 1 — Table S1: HLA class I genotypes of the autologous and allogeneic donors. [file CPR-9999-e70261-s001.docx]

**Supplementary Table 1. HLA class I genotypes of the autologous and allogeneic donors**

| Locus | Autologous Donor (iPSC source) | Allogeneic Donor |
| --- | --- | --- |
| HLA-A | 24:02, 33:03 | 11:01, 24:02 |
| HLA-B | 54:01, 58:01 | 15:01, 40:01 |
| HLA-C | 01:02, 03:02 | 01:02, 07:02 |
